# A Secret Revealed: The Coexistence of a Pheochromocytoma and Independent Adrenocorticotropic Hormone-Secreting Cushing Syndrome Within the Same Adrenal Gland

**DOI:** 10.7759/cureus.83943

**Published:** 2025-05-12

**Authors:** Sana Rafi, Meryam Alahyane, Ghizlane Elmghari, Nawal El Ansari, Fatim Zahra Hazmiri, Oumayma Ait Ouhssain, Hanane Rais

**Affiliations:** 1 Endocrinology, Diabetes and Metabolism, University Hospital Mohammed VI, Marrakech, MAR; 2 Biosciences and Health Laboratory, Faculty of Medicine and Pharmacy of Marrakech, Marrakech, MAR; 3 Pathology, University Hospital Mohammed VI, Marrakech, MAR; 4 Morphosciences Research Laboratory, Faculty of Medicine and Pharmacy of Marrakech, Marrakech, MAR

**Keywords:** acth, adrenal incidentaloma, adrenal pathology, cushing’s syndrome, pheochromocytoma

## Abstract

Adrenal incidentalomas are increasingly encountered in clinical practice. When functional, they are typically associated with the secretion of a single hormone. However, dual hormonal activity is exceptionally rare, particularly in cases where pheochromocytomas are associated with adrenocorticotropic hormone (ACTH) production, as seen in ectopic Cushing syndrome. We report a unique case of a single adrenal mass, discovered incidentally during a thoracic CT scan performed as part of post-COVID follow-up. Biochemical investigations revealed elevated 24-hour urinary metanephrines, a non-suppressible cortisol level after an overnight dexamethasone suppression test, and normal ACTH levels, suggesting an atypical secretory profile. Histopathological examination confirmed the mass as a pheochromocytoma. Notably, clusters of ACTH-expressing cells were found surrounding the tumor. These cells did not form a distinct mass and were interpreted as part of the same lesion. It has been hypothesized that they may correspond to Leydig-like cells resulting from an embryological migration defect. Additionally, a paracrine interaction between chromaffin cells and ACTH-expressing cells, potentially mediated by co-peptides, may contribute to this unique hormonal behavior. This case adds to the exceptionally rare reports of dual hormonal activity in adrenal tumors and offers new insights into the pathophysiological mechanisms underlying this unusual endocrine presentation.

## Introduction

Adrenal incidentaloma is a tumor whose incidence has increased in recent years due to the widespread use of CT imaging [1]. In most cases, these tumors are benign and non-functional; only 10% are functional. When functional, they are typically pheochromocytomas or cases of ACTH-independent hypercortisolism. More rarely, they may involve an ACTH-secreting pheochromocytoma in the context of ectopic Cushing syndrome [2]. Here, we report an exceptional case of an adrenal incidentaloma characterized by the coexistence of a pheochromocytoma and autonomous cortisol secretion by cells expressing ACTH independently of the adrenal cortex.

## Case presentation

A 68-year-old woman was referred to our institution for an etiological workup of a right adrenal incidentaloma, discovered during a thoracic CT scan performed for suspected COVID-19 infection. Her medical history included long-standing type 2 diabetes mellitus managed with insulin therapy, hypertension controlled with dual therapy, and a history of lumbar disc herniation under follow-up. She reported generalized fatigue, difficulty climbing stairs, and walking long distances but denied weight gain or clinical signs of Cushing syndrome.

On physical examination, she had obesity (BMI: 34 kg/m²), central adiposity (waist circumference: 118 cm), and slender lower limbs, with no striae or signs of skin fragility. An abdominal CT scan revealed a 27 × 26 mm right adrenal nodule with a rounded shape, regular contours, and heterogeneous density, including a cystic component. The tumor had a spontaneous density of 33 Hounsfield units (HU) and a washout greater than 50%.

Biochemical analysis showed normal potassium levels (4 mmol/L), a negative 1-mg dexamethasone suppression test (post-test cortisol: 1.9 µg/dL), and a borderline 8 am ACTH level (12 pg/mL), suggesting ACTH independence. Additionally, 24-hour urinary metanephrines and normetanephrine were elevated, reaching twice the upper limit of normal (Table 1). Given these findings, initial management consisted of surveillance with deferred surgery.

**Table 1:** Hormonal and biochemical evaluation of the patient throughout follow-up

		Normal range	On hospital admission	After six months of follow-up
Baseline 8 am ACTH		Cutoff value: 15 pg/mL	12 pg/mL	-
24-hour urinary free cortisol		4.3-176 μg/24 hours	6.75	-
Morning cortisol after 1 mg overnight dexamethasone		<1.8 μg/dL	1.9	3
24-hour urinary metanephrines and normetanephrines	Metanephrine	0.04-0.18 mg/24 hours	0.38	0.74
	Normetanephrine	0.07-0.38 mg/24 hours	0.80	1.40
	3-methoxytyramine	<55 ug/24 hours	23	
Potassium		3.5-4.5 mEq/L	4	4.1

**Figure 1:**
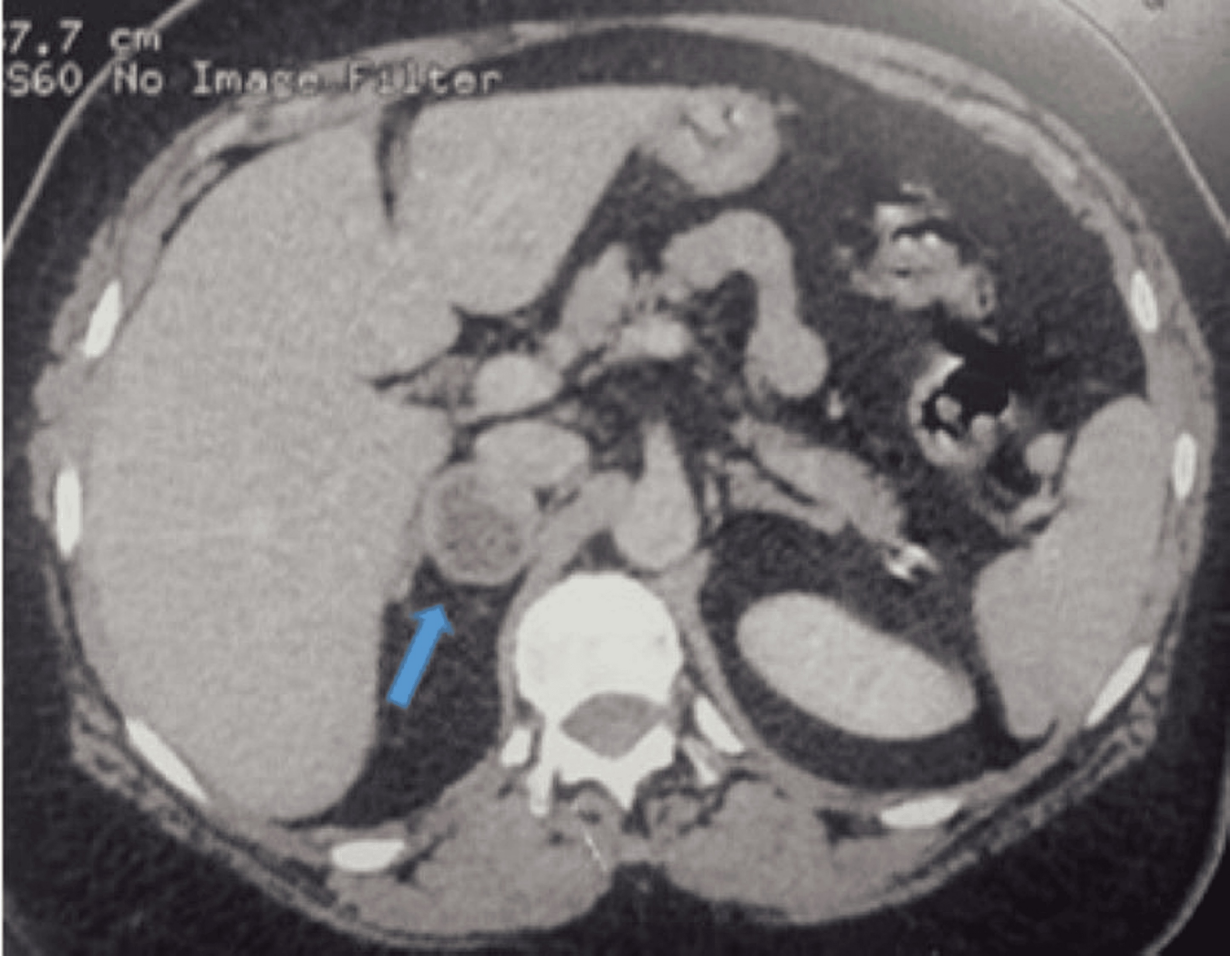
Adrenal scan in axial cross-section. Blue arrow: Right adrenal mass involving the medial limb, measuring 30.4 × 29 mm, rounded with regular contours, heterogeneous density with a cystic area. Spontaneous density = 33 HU; absolute washout > 50%.

At the six-month follow-up, repeat adrenal CT showed an increase in tumor size to 30.4 × 29 mm (Figure 1).

Hormonal reassessment revealed a persistently negative 1-mg dexamethasone suppression test (post-test cortisol: 3 µg/dL) and a fourfold increase in urinary metanephrines (Table 1).

Based on these findings, the diagnosis of an ACTH-secreting pheochromocytoma was established. The patient underwent preoperative alpha-blockade and rehydration before laparoscopic adrenalectomy.

**Figure 2:**
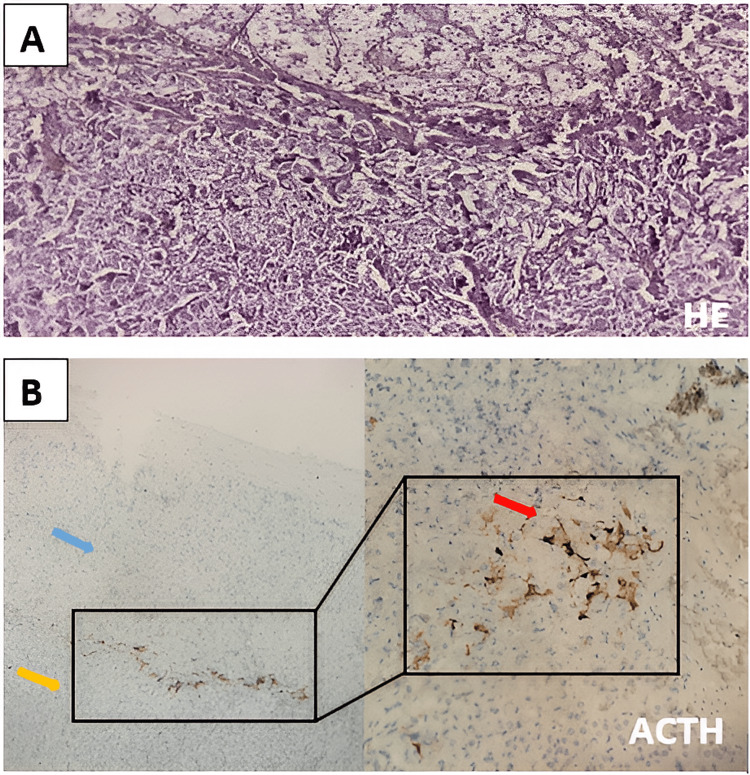
Histological images of slides stained with hematoxylin and eosin (A); immunohistochemistry images (B). (A) Tumor proliferation arranged in "zellballen" nests outlined by sinusoidal vascularization with residual adrenal cortical parenchyma (top) (H&E ×10). (B) Blue arrow: residual adrenal parenchyma; yellow arrow: pheochromocytoma; red arrow: cytoplasmic expression of the anti-ACTH antibody in a few isolated cells or clusters, observed in the transition zone between the residual adrenal parenchyma and pheochromocytoma. ACTH, adrenocorticotropic hormone

The postoperative course was uneventful. Histopathological examination confirmed the presence of a pheochromocytoma measuring 2 cm, with a Pheochromocytoma of the Adrenal Gland Scaled Score (PASS) score of 3 (Figure 2A). Immunohistochemical staining for ACTH within the tumor was negative, but clusters of ACTH-expressing cells were identified in the peritumoral region, suggesting ACTH-independent cortisol secretion (Figure 2B). Postoperative biochemical assessment showed normal 24-hour urinary metanephrines and normetanephrines, along with a positive overnight dexamethasone suppression test.

## Discussion

The incidence of adrenal incidentalomas has increased due to the widespread use of imaging, with an estimated prevalence of 2-4% in abdominal CT scans [1]. While most are benign and non-functional, approximately 10% exhibit hormonal activity [1,2]. Our case is noteworthy due to the functional nature of the incidentaloma, which presented a dual secretory profile. This led us to hypothesize the presence of an ACTH-secreting pheochromocytoma within the context of ectopic Cushing syndrome.

This rare entity has been described in the literature, with most reported cases exhibiting overt Cushing syndrome, elevated ACTH levels, and positive ACTH immunostaining within the tumor [3-5]. However, in our case, Cushing syndrome was subclinical, with normal ACTH levels and negative intra-tumoral ACTH immunostaining. The identification of ACTH-expressing peritumoral cell clusters suggests an alternative mechanism of ACTH-independent hypercortisolism associated with a pheochromocytoma.

Several hypotheses have been proposed to explain this phenomenon. One possibility is the expression of ectopic hormone receptors, akin to macronodular adrenal hyperplasia, which can lead to subclinical ACTH-independent Cushing syndrome [6-8]. Another plausible explanation lies in the embryological origin of the adrenal gland. The adreno-gonadal primordium gives rise to both the adrenal cortex and gonadal structures [9]. The presence of ACTH-expressing peritumoral cells may represent Leydig-like cells that failed to migrate properly during embryogenesis [7,9]. Furthermore, paracrine interactions between chromaffin cells and co-secreted peptides have been implicated in the modulation of adrenal steroidogenesis [7,10,11].

Unlike previously described cases of ACTH-secreting pheochromocytomas, our case does not fit this classification. Instead, it represents the coexistence of a pheochromocytoma and an independent source of ACTH-expressing cells driving autonomous cortisol secretion.

## Conclusions

This case contributes to the growing body of exceptional observations in adrenal pathology and highlights the importance of a nuanced approach to adrenal incidentalomas with discordant biochemical findings. While ACTH-secreting pheochromocytomas are well-established causes of ectopic Cushing syndrome, our findings support the hypothesis of a novel mechanism involving paracrine interactions between pheochromocytomas and peritumoral ACTH-expressing cells. This possibility introduces a previously underexplored dimension of adrenal endocrine regulation. Future studies should investigate the immunohistochemical characterization of peritumoral ACTH expression and the molecular basis of paracrine signaling in functional adrenal tumors, with potential implications for diagnosis and management.
